# Percutaneous endoscopic cecostomy for management of Ogilvie’s syndrome: a case series and literature review with an update on current guidelines (with video)

**DOI:** 10.1007/s00464-023-10281-w

**Published:** 2023-07-27

**Authors:** Petr Vanek, Ondrej Urban, Premysl Falt

**Affiliations:** 1 Faculty of Medicine and Dentistry, Palacky University Olomouc, Hnevotinska 976/3, 77515 Olomouc, Czech Republic; 2 Department of Internal Medicine II – Gastroenterology and Geriatrics, University Hospital Olomouc, Olomouc, Czech Republic

**Keywords:** Ogilvie’s syndrome, Acute colonic pseudo-obstruction, Endoscopic decompression, Percutaneous endoscopic cecostomy, Colostomy, Case series

## Abstract

**Introduction:**

Percutaneous endoscopic cecostomy (PEC) is a viable treatment option for patients with persistent or recurrent acute colonic pseudo-obstruction (ACPO; Ogilvie’s syndrome). It should be generally considered in patients that are refractory to pharmacologic and endoscopic decompression, especially those not amenable to surgical intervention due to an increased perioperative risk. Physicians are rather unfamiliar with this approach given the limited number of reports in the literature and paucity of guideline resources, although guidelines concerning ACPO and covering the role of endoscopy were recently published by three major expert societies, all within the last 2 years.

**Patients and methods:**

We retrospectively identified three consecutive patients who underwent PEC placement at a Czech tertiary referral center between May 2018 and December 2021: all for recurrent ACPO. In addition, we summarized the current guidelines in order to present the latest knowledge related both to the procedure and management approach in patients with ACPO.

**Results:**

The placement of PEC was successful and resulted in clinical improvement in all cases without any adverse events.

**Conclusion:**

The results of our experience are in line with previous reports and suggest that PEC may become a very useful tool in the armamentarium of modalities utilized to treat ACPO. Furthermore, the availability of guideline resources now offers comprehensive guidance for informed decision-making and the procedural aspects.

**Supplementary Information:**

The online version contains supplementary material available at 10.1007/s00464-023-10281-w.

Acute colonic pseudo-obstruction (ACPO), more commonly known as Ogilvie’s syndrome, refers to pathologic dilatation of the colon without underlying mechanical obstruction [1]. It occurs primarily in elderly, hospitalized, or institutionalized patients with comorbid conditions, infection, metabolic imbalance, or who are recovering from surgery or trauma (Table 1) [2–4]. The precise pathogenesis is unknown, but evidence suggests an alteration in the autonomic nervous system resulting in colonic atony [5, 6]. A United States-based retrospective cohort study estimated the incidence of ACPO to approximately 100 cases of 100,000 hospital admissions per year [7]. The main clinical features include acute massive abdominal distention and pain along with nausea, vomiting, and altered bowel function [8–10]. The diagnosis is established by abdominal imaging using computed tomography (CT) or water-soluble contrast enema, showing colonic dilatation without evidence of mechanical obstruction [11, 12].

**Table 1:** Main conditions associated with Ogilvie’s syndrome (according to [2–4])

Category	Examples
Medication	Opioids, anticholinergics, Ca^++^ channel blockers, laxative abuse, cytotoxics, antipsychotics, benzodiazepines, dopaminergics, epidural anesthesia
Trauma and Orthopedics	Pelvic and hip fracture, hip and spine surgery, burns
Cardiopulmonary	Cardiac surgery, mechanical ventilation, myocardial infarction, pneumonia, congestive heart failure, chronic obstructive pulmonary disease
Neurologic	Parkinsonism, stroke, dementia, multiple sclerosis
Oncologic	Small cell lung cancer, multiple myeloma, acute myeloid leukemia, pelvic irradiation, retroperitoneal invasion, disseminated malignancy
Obstetrics	Pregnancy, normal or instrumental delivery, cesarean section, hysterectomy
Metabolic	K^+^, Ca^++^, Mg^++^ imbalance, diabetes, hypothyroidism
Infectious	Sepsis, herpes zoster, cytomegalovirus
Inflammation	Appendicitis, cholecystitis, pancreatitis, gastritis

The goal of management is to decompress the distended colon to minimize the risk of mural ischemia and colonic perforation, both associated with high mortality [2]. Supportive measures remain the first-line step and include identification and discontinuation of precipitating factors (e.g., medication), correcting fluid and electrolyte disturbances, maintaining patients with *nil per os*, decompressing the GI tract by nasogastric or rectal tubes, and frequent position changes [10–12]. In case the condition is not improving with supportive care, both medical therapy with neostigmine and decompressive colonoscopy are considered valid options [12–14].

In patients with ACPO in whom supportive, pharmacologic, and conventional endoscopic therapies fail, percutaneous endoscopic cecostomy/colostomy (PEC) may be considered before proceeding with surgical interventions [15, 16]. The method was first described by Ponsky and colleagues (1986) as an alternative to surgically or radiographically placed colostomy in two patients who were unsuccessfully treated with decompressive colonoscopy [17]. Since then, it has been gradually extending into practice, although its widespread use has been limited. Successful utilization of PEC requires local familiarity with the method, cautious indication, available expertise, and multidisciplinary support. However, our experience indicates that treating physicians and/or endoscopists are generally not acquainted with the procedure, perhaps because of its rarity due to the few defined indications, infrequent occurrence in the literature, and a relative paucity of guideline resources given the fact that major endoscopic and surgical societies published applicable recommendations just recently.

We report a case series of patients with ACPO who were ultimately managed with PEC placement after traditional therapeutic approaches had failed. In addition, we describe our experience in the context of the literature and the newly published recommendations, providing guidance on the therapeutic approach in ACPO along with indications, techniques, and periprocedural management.

## Patients and methods

We identified three consecutive patients who underwent PEC placement at a Czech tertiary referral center: all for recurrent ACPO. The procedures were performed between May 2018 and December 2021 and are presented in chronological order as a single-center retrospective case series, reported in line with the PROCESS 2020 Guideline [18]. The interventions were performed using a modification of the technique initially described by Ponsky and colleagues, which was originally derived from the pull-through variant of percutaneous endoscopic gastrostomy (PEG) [17]. Given that all patients presented with ACPO, no oral bowel preparation was attempted, but the patients were kept fasted for at least 12 h prior to the procedure. Intravenous antibiotics (amoxicillin/clavulanic acid) were administered prophylactically before the procedure to provide coverage against both gram-negative (fecal) and gram-positive (skin) bacteria, and they were continued for 24 h post-intervention.

**Fig. 1:**
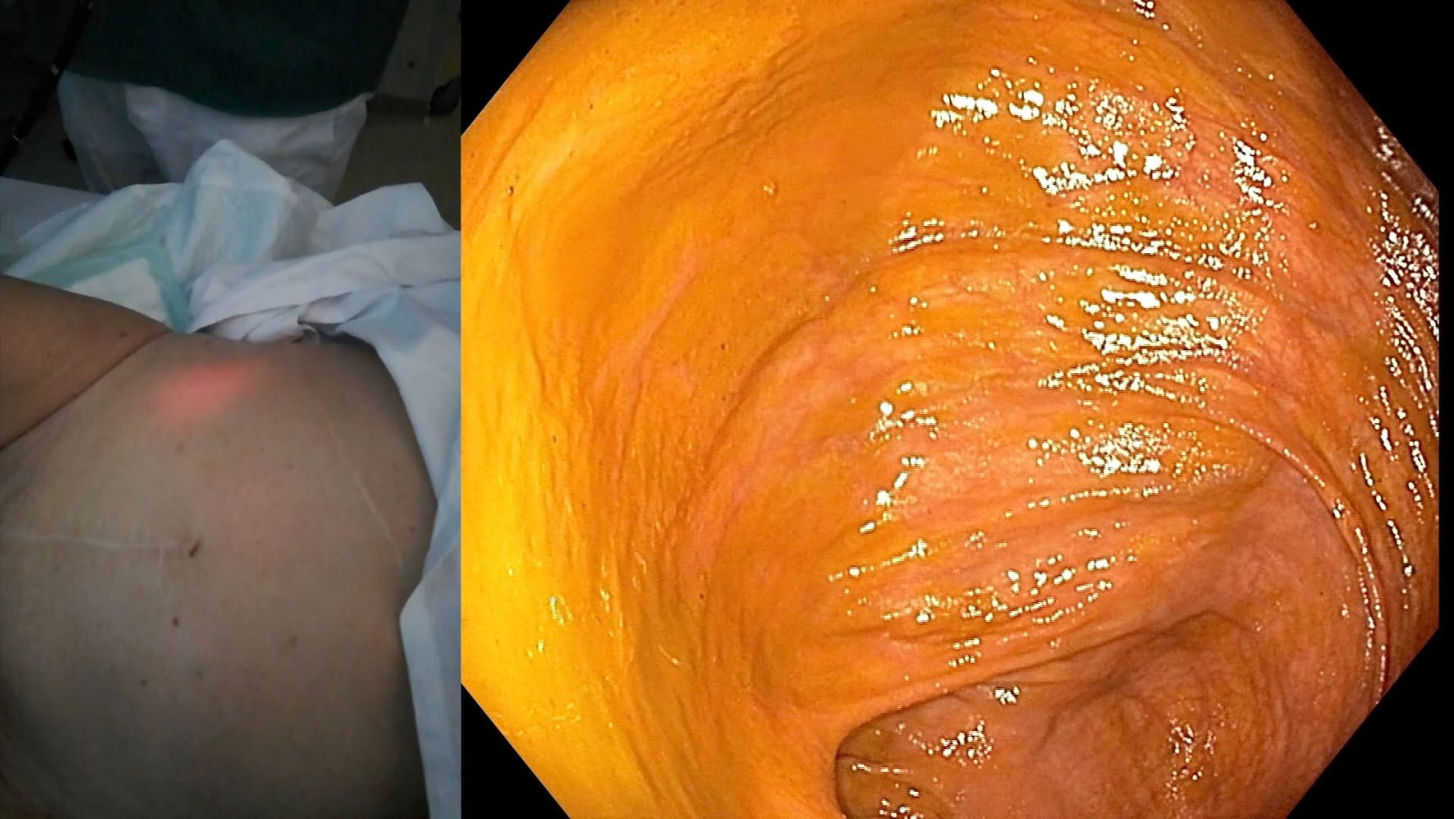
Transillumination of the abdominal wall (left); endoscopic view within the right colon (right)

**Fig. 2:**
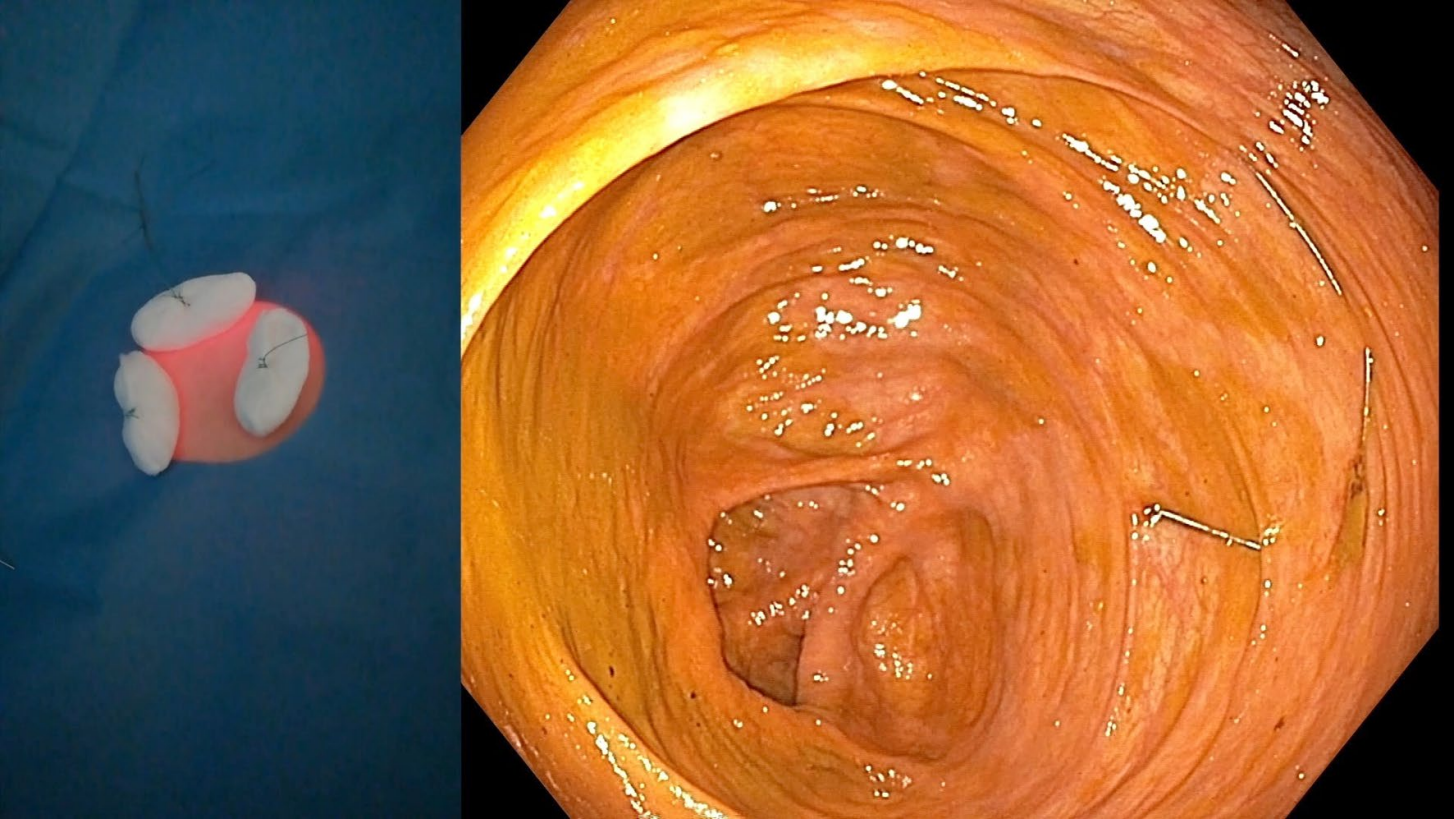
Three anchoring sutures secured onto tampons fixing the colon to the anterior abdominal wall (left); endoscopic view of the cecopexy site (right)

**Fig. 3:**
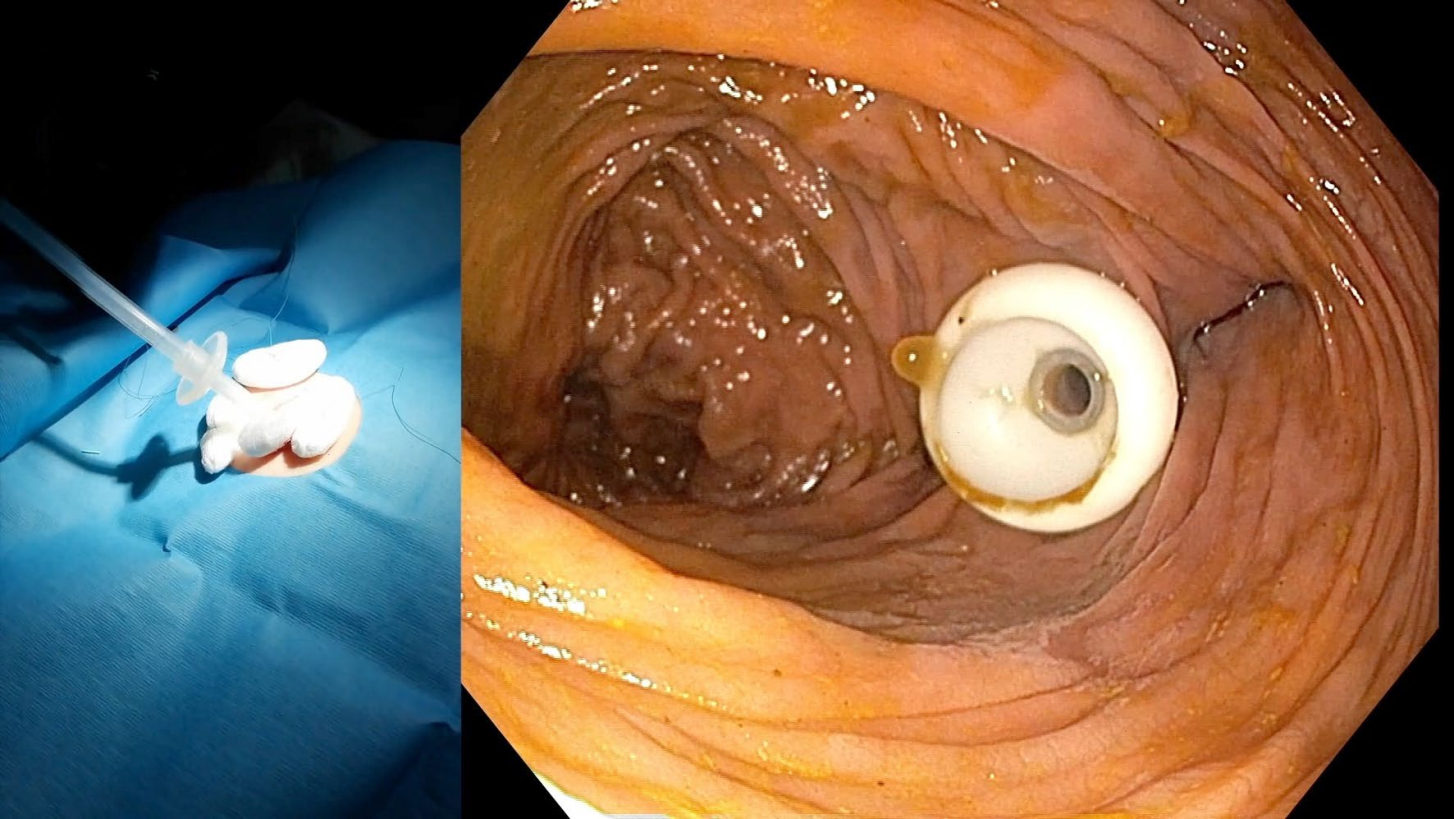
Fastening the cecostomy tube with the external bumper (left); endoscopic view confirming the correct position of the internal bumper after colonoscope reinsertion (right)

The procedures were conducted with the patient lying supine and under general anesthesia in case 1, whereas cases 2 and 3 involved conscious sedation utilizing titrated doses of midazolam, with the active participation of two operators. All three cases were managed by a consistent team of two skilled advanced endoscopists. Refer to Video 1 (Online Supplementary Material), which offers a detailed demonstration of all procedural aspects from both endoscopic and external viewpoints. First, a standard adult colonoscope was advanced into the right colon with transillumination of the abdominal wall to identify a suitable puncture site (Fig. 1). Notably, appropriate attention was devoted to keep CO_2_ insufflation at minimum to avoid the risk of perforation of the already distended colon. Following intubation of the cecum, the adjacent lumen was cleansed to remove fecal material. The proper position was confirmed by an indentation of the cecum with direct digital pressure on the abdominal wall. The abdominal wall was then prepared with betadine solution, anesthetized (1% trimecaine), and draped in a sterile fashion. The contents of a standard, commercially available PEG kits with 24F tubes and an anchor suture system (PEG-24-PULL-I-S and Cope Gastrointestinal Suture Anchor Set, Cook, Inc., Bloomington, IN, USA) were subsequently used. Provided that sufficient transillumination was achieved, a dedicated kit needle preloaded with the anchor system was introduced through the abdominal wall into the cecum. An intracolic position was confirmed by direct visualization and aspiration of air into a syringe. A stylet wire was then passed through the needle, advancing a T-anchor into the colon. The wire and needle were then removed, and the colon apposed anteriorly to the abdominal wall by gentle traction on the anchor suture. The cecum was gradually fixated using a total of three T-anchors in a triangular configuration approximately 3 cm apart (Fig. 2). The center of the cecopexy site was then punctured with a 19-gauge Seldinger needle. A traction wire was passed through the needle, grasped by a snare, and then withdrawn from the colon along with the colonoscope. The remainder of the procedure was analogical as for the insertion of PEG using the pull technique, with the tube slowly trailed through the anus, colon, and abdominal wall. The correct position was confirmed by reinsertion of the colonoscope (Fig. 3). Simultaneously, the wound site was covered with sterile dressing, and the tube was fastened by an external bumper. The T-anchors were removed 1–2 weeks later.

From the therapeutic point of view, the PEC tube was left open for the first 24 h to allow venting. After that, the tube was capped and regularly flushed with 30–60 mL of tap water every 8 h to keep it patent. In addition, it was opened 2–4 times daily for decompression based on the course of the condition in each patient.

## Case series

The patient and procedure characteristics including outcomes are summarized in Table 2. Notably, case 1 was also reported as the first procedure of its kind in the Czech Republic [19].

**Table 2:** Summary of patient and procedure characteristics with PEC outcomes

Case	Age (years)/gender	Indication	Anesthesia	Site of PEC	Tube size	Adverse events	Outcome	Follow-up
1	89/F	ACPO	General anesthesia	Cecum	24F	None	ACPO resolved; left in place*	15 months
2	67/M	ACPO	Conscious sedation	Ascending colon	24F	None	ACPO resolved; left in place**	2 months
3	66/M	ACPO	Conscious sedation	Cecum	24F	None	ACPO resolved; removed at 3 months	12 months

*PEC* percutaneous endoscopic colostomy, *ACPO* acute colonic pseudo-obstruction

*PEC was used intermittently for decompression and irrigation until the patient’s death

**Patient succumbed to underlying malignancy 2 months after PEC placement

### Case 1

**Fig. 4:**
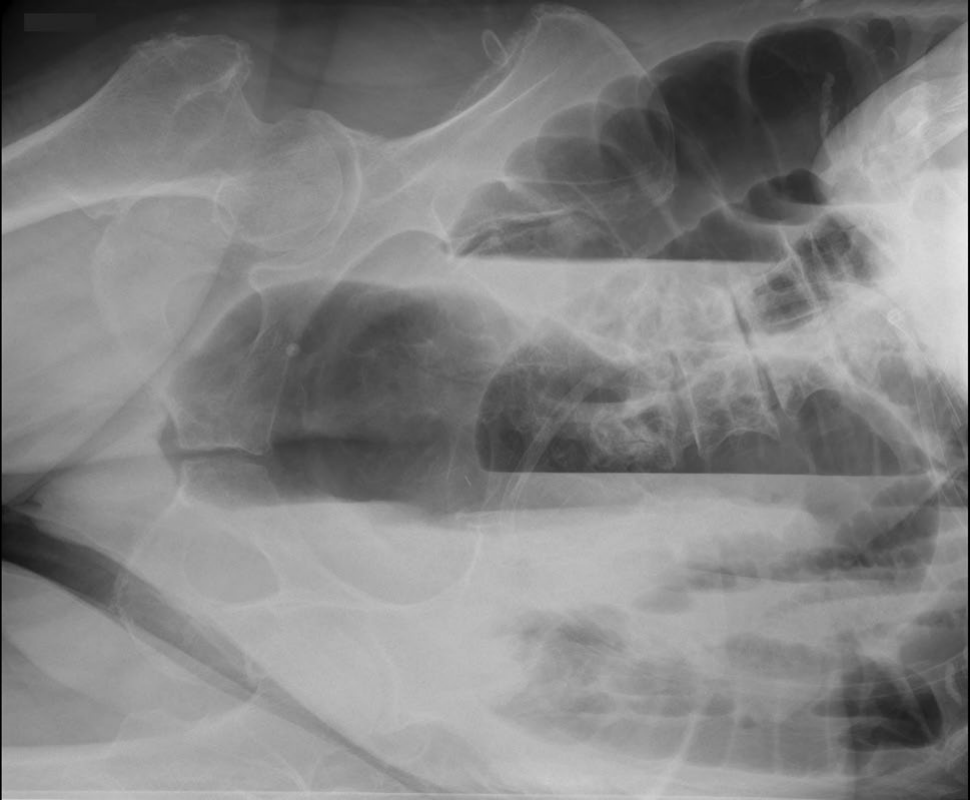
Left lateral decubitus radiograph shows gas filling the entire colon down into the rectosigmoid in a patient with marked colonic dilatation

**Fig. 5:**
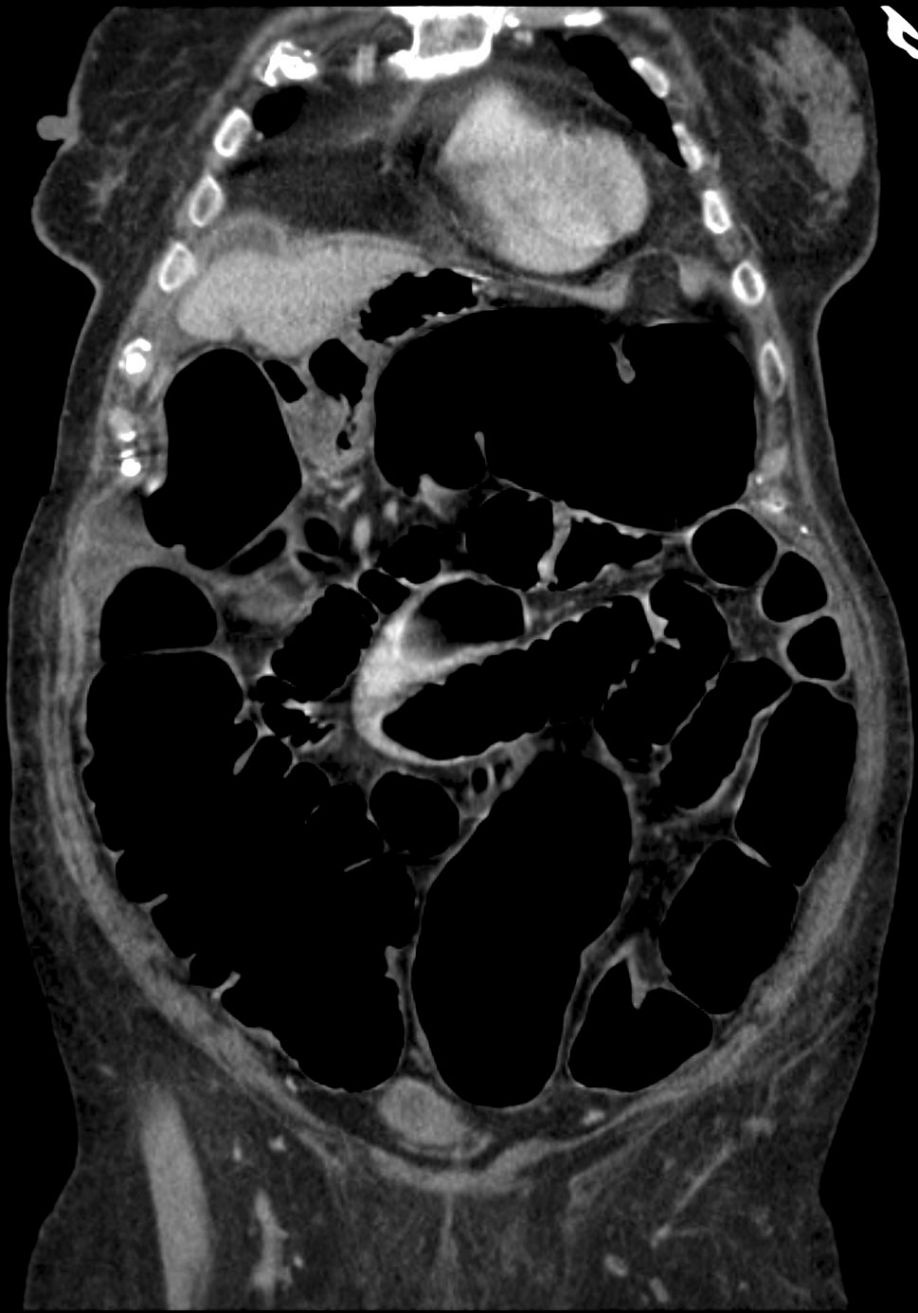
Abdominal CT scan demonstrating small bowel distention and diffuse dilatation of the large bowel without evidence of any abrupt transition point or mechanically obstructing lesion

An 89-year-old immobile female with an extensive past medical history including two ischemic strokes, multiple fractures due to falls at home, and dementia was referred for severe abdominal pain, vomiting, and dehydration. The initial abdominal radiograph showed marked dilatation of the colon with multiple air-fluid levels (Fig. 4), while laboratory analysis did not reveal any significant or pathognomonic findings. Abdominal CT was performed, excluding mechanical obstruction or other organic pathology (Fig. 5). The patient was diagnosed with ACPO and admitted for conservative treatment. Nevertheless, comprehensive care including maximum pharmacotherapy with neostigmine did not bring sufficient clinical effect. Decompressive colonoscopy was necessary, although ACPO recurred within a few days necessitating repeated colonoscopies. Given that conventional therapy had failed, surgical colostomy with possible subtotal colectomy as the utmost option was proposed, but the idea was abandoned because of poor surgical candidacy. Finally, PEC was performed without any adverse events and with immediate effect, prompting patient discharge to a nursing home a week later. The cecostomy functioned well and had been used intermittently as a decompression and irrigation canal for the next 15 months, at which time the patient succumbed to natural causes.

### Case 2

A 67-year-old male with generalized salivary gland carcinoma and multiple comorbid conditions developed severe obstipation with abdominal distension, necessitating protracted use of enemas and laxatives. The condition gradually progressed to the point when the patient required hospitalization and colonoscopic decompression, which had only a temporary effect lasting 48 h. It was established as a paraneoplastic case of ACPO, and the patient was indicated for PEC by a multidisciplinary team decision. After the procedure the symptoms of intestinal obstruction improved and bowel movements were restored, however the patient succumbed to his malignancy within 2 months. Of note, the patient had the colostomy tube placed in the proximal part of the ascending colon given that pseudomembranes and underlying trophic changes of the cecal mucosa were noted during the procedure. Moreover, his cecum was localized in an atypical position and transilluminated above the umbilicus.

### Case 3

A 66-year-old obese male with a body mass index of 35 was initially admitted due to acute epididymitis. The hospital stay was complicated by gentamicin-associated acute renal injury, which confined the patient to bed for extended time and resulted in ACPO. Conservative management was not successful, and the patient was referred to our department for endoscopic decompression. Two decompressive colonoscopies were performed but resolved symptoms only for 2 and 3 days, respectively. Being a poor operative candidate, the patient required PEC placement. Cecostomy provided immediate decompression and led to durable symptom relief without any adverse events, after which the patient was discharged. The tube was sealed 8 weeks after placement in the absence of further need for decompression, given that the patient had restored mobility and regular defecation. The tube was removed 3 months after placement by cutting it at the skin level and pushing it into the colon under endoscopic visualization, after which it was extracted using a snare. The fistula tract healed uneventfully within 2–3 weeks, and the patient was without any symptoms of colonic distention at 12 months.

## Current guidelines

**Fig. 6:**
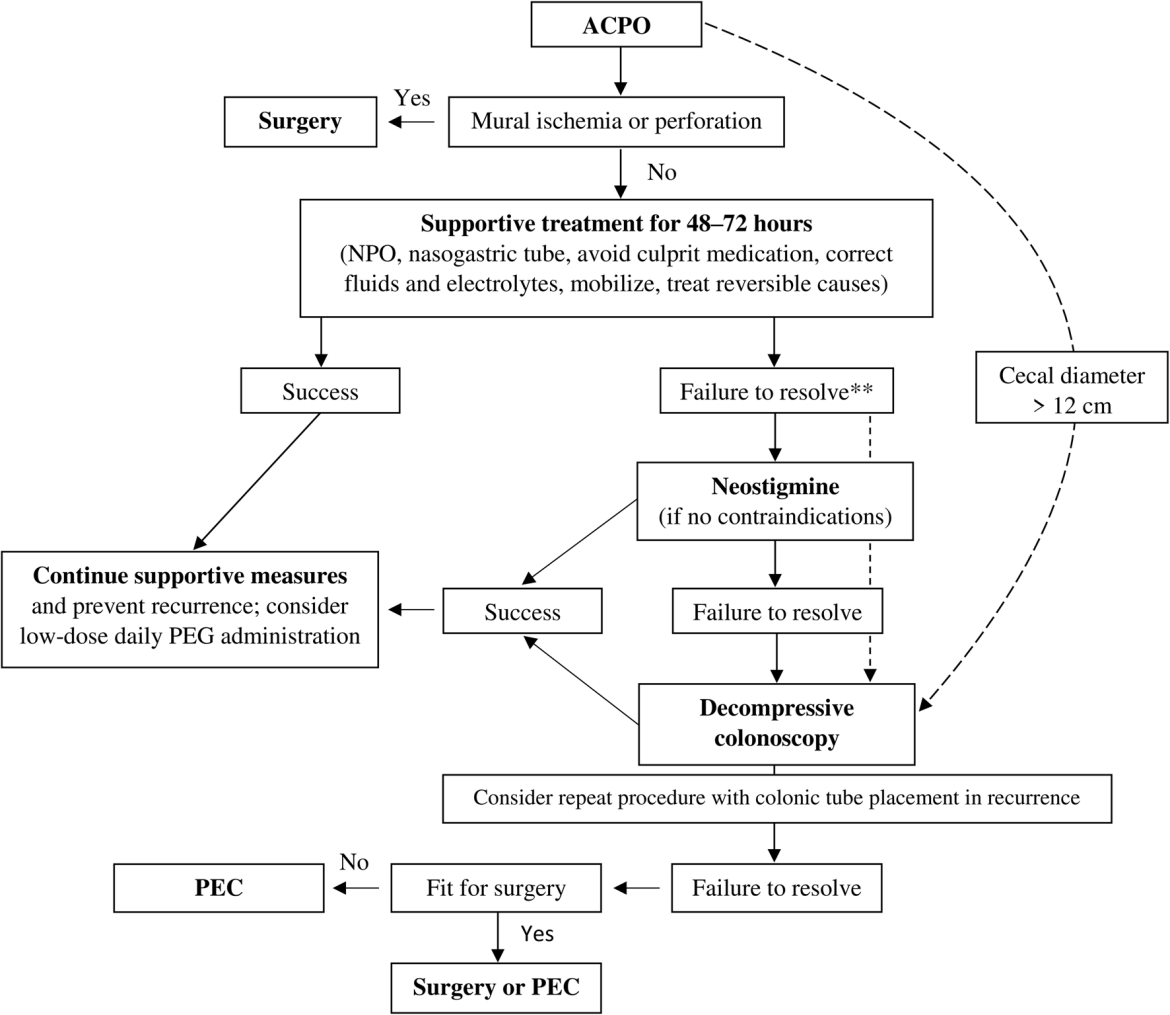
Recommended treatment algorithm in patients with ACPO (adapted according to [12–14]). *ACPO* acute colonic pseudo-obstruction, *NPO* nil per os, *PEG* polyethylene glycol, *PEC* percutaneous endoscopic cecostomy. **No recommendation can be made for the superiority of neostigmine or decompressive colonoscopy as better initial therapy at the time; the choice of treatment strategy should also depend on local expertise and situation

Guidelines concerning ACPO and covering the role of endoscopy were recently published by the European Society of Gastrointestinal Endoscopy (ESGE), the American Society for Gastrointestinal Endoscopy (ASGE), and the American Society of Colon and Rectal Surgeons (ASCRS), all within the last 2 years [12–14]. Figure 6 outlines the recommended treatment algorithm.

Baseline laboratory analysis, including complete blood count, metabolic panel, and thyroid hormones, should be performed during the initial evaluation to look for predisposing and treatable causes [12, 14]. Supportive care remains the first-line therapy in patients with uncomplicated course, i.e., absence of ischemia or peritonitis and cecal diameter < 12 cm. It comprises maintaining the patient with *nil per os*, fluid resuscitation, correcting electrolytes, nasogastric or rectal tube insertion, ambulation, treatment of underlying disease, and avoidance of culprit medication [12–14]. The pharmacologic agent of choice is neostigmine, a short-acting cholinesterase inhibitor, and it is indicated in patients in whom symptoms persist beyond 48–72 h despite supportive care [12–14]. Contraindications to its use include evidence of intestinal or urinary obstruction and cardiopulmonary monitoring with immediate access to atropine in the event of bradycardia is recommended [13, 14]. Neostigmine is typically administered intravenously in a single bolus of 2–5 mg, with a second dose reserved for initial non-responders or partial responders. However, a consensus on dosing does not exist and routes of administration vary [4, 13, 14].

Nonoperative methods of colonic decompression are generally recommended in patients who have failed supportive measures, are not candidates for conservative management, or in whom neostigmine is contraindicated or ineffective [12–14]. Notably, as there are no prospective head-to-head trials between decompressive colonoscopy and neostigmine therapy, no recommendation can be made for the superiority of one of these options; the choice of treatment strategy should also depend on local expertise and situation, e.g., access to urgent colonoscopy [12]. Nonetheless, prompt endoscopic decompression by means of colonoscopic desufflation is recommended in cecal diameter > 12 cm or if the syndrome exists longer than 4–6 days [12]. The risk of ACPO recurrence after successful decompression varies widely in the literature, ranging from 0 to 50% [20, 21], and repeated endoscopic decompressions with colonic tube placement as an alternative may be considered in these cases [12].

Patients that are refractory to pharmacologic and endoscopic decompression, especially those not amenable to surgical intervention due to an increased perioperative risk, should be considered for PEC [12, 14]. The necessary steps include good bowel cleansing, use of sedation, disinfection of the abdominal wall, transillumination, and fixation of the colon to the abdominal wall [12]. As mortality rates are substantial in ACPO patients requiring surgical interventions, all efforts should be made to manage this patient population nonoperatively [13]. Surgery is the most appropriate step in cases complicated by peritonitis, colonic ischemia, and/or perforation or when all other options fail, and it includes surgically placed cecostomy tube, colostomy, or subtotal colectomy [13, 14].

## Discussion

Management of patients with ACPO is challenging, with the goal of maximal mitigation of symptoms and prevention of potential adverse events associated with the condition. The effectiveness of pharmacologic and endoscopic therapy has reduced the need for surgery to cases complicated by colonic ischemia, perforation, or colonic dilatation otherwise refractory to nonoperative options [4, 8, 10, 20, 22]. The method of PEC serves as an important alternative especially in patients that are unfit for surgery [12, 23]. The procedure has evolved from the method of PEG and was first described in 1986 [17]. Nonetheless, its widespread use has been limited with scarce literature regarding technique, efficacy and safety, seldom indications, and a lack of awareness among physicians.

In general, studies on PEC are sparse and data collection is retrospective. Based on a recent systematic review by Khayyat on therapeutic utility of PEC in adults, the existing evidence is heterogeneous and consists mainly of case reports and case series [24]. Up until 2020 the dedicated guideline resource on PEC placement had been the recommendations by the National Institute for Health and Clinical Excellence published in 2006 [25]. However, according to the evidence used to support this guidance, the literature review was limited to one case series and three case reports. Several more studies reported data regarding PEC in various indications since then, however, the total number of patients with ACPO remains relatively low. Baraza and colleagues performed 35 PECs in 33 patients, of whom 4 had recurrent ACPO and were considered poor surgical candidates [26]. Symptoms resolved in 26 patients (74%), including 3 of the 4 with ACPO. Major adverse events occurred in 4 patients (3 had peritonitis secondary to fecal contamination; 1 patient died). Another study by Lynch and colleagues reported data on PEC placement in 8 patients: 6 for ACPO and 2 for chronic constipation [16]. Seven of the 8 cases were successful and resulted in clinical improvement, 1 patient required surgical removal of the PEC tube for fecal spillage resulting in peritonitis. Cowlam and colleagues reported their experience with an improvement in symptoms in 81% of 31 patients treated with PEC, including 5 patients with ACPO [27]. Nonetheless, 3 of these 5 patients had to have the tube removed because of infection and 1 patient died from fecal peritonitis. Similar outcomes were reported by other authors [15, 17, 28].

Conveniently regarding the aforementioned, dedicated guidelines covering the role of PEC in the management of ACPO were recently published by three major societies. The aim within the ESGE guideline was for the first time to provide guidance on the technique and management of PEC placement in patients with intractable constipation and ACPO [12]. The ASGE document updated on the role of endoscopy in the management of colonic volvulus and ACPO [13]. The ASCRS guideline focused on the evaluation and treatment of sigmoid or cecal volvulus and ACPO [14].

Indications for the procedure include intermittent or continuous decompression in refractory ACPO, colon fixation in recurrent colonic volvulus, and antegrade colonic enema in selected cases of evacuatory disorder or constipation not responding to other options [12–14, 25]. Oral bowel preparation should not be used in ACPO as it may worsen colonic dilatation in the absence of bowel transit [12, 22]; however, it is recommended in cases of intractable constipation as abundant fecal remnants might increase the risk of septic adverse events [12]. Even though there has been no study addressing the need for antibiotics, they have been administered in general practice in view of the potential fecal contamination [15, 16, 29–32]. According to the ESGE guideline, antibiotic prophylaxis should follow local protocols and start 1 h prior and continue for 3 days post-procedure [12]; the other guidelines did not report on this [13, 14, 25]. In addition, the procedure should be performed with CO_2_ insufflation and the patient in the left lateral or supine position.

The cecum is the preferred location of PEC placement unless it is technically not feasible [12, 13]. While it is possible to perform colostomy at other locations, there are no data demonstrating any advantages [26, 27, 33, 34]. Furthermore, cecal transillumination is a prerequisite to increase safety and procedure ease as it helps in determining the most direct route into the colon. Cecostomy also allows pancolonic rather than distal enema in order to provide more effective bowel function [35]. Notwithstanding, sigmoid volvulus is a specific situation in which reported techniques vary in terms of the number and site of fixation [13, 14]. Some authors suggest inserting colostomy in two locations, preferably on both limbs of the volvulus to fix the colon and prevent further “twisting” [23].

Three main techniques have been used in clinical practice: the pull-through method, the introducer (“push”) method, and laparoscopically assisted PEC (LAPEC) [16, 26, 29, 32, 36, 37]. The existing data do not provide evidence as to which method should be preferred, and the method itself has not been standardized [12–14]. Surprising differences exist regarding the type and size of tubes, which is likely to play a role in the efficacy and tolerability of the system [24]. Nonetheless, ESGE recommends fixing the colon to the abdominal wall at three points using T-anchors, a double-needle suturing device, or laparoscopic fixation, whatever method is used [12]. This is key in ensuring a stable colon position during the procedure to prevent leaks. The technical success rates of solely endoscopic cecostomy surpass 80%; adverse events occur in 30–40%, and quality of life improves for most patients, even though acceptance is reduced in approximately 25% of patients, mostly because of pain [12, 15, 16, 27, 29, 30, 38–43]. Although most adverse events are minor, mortality has been noted secondary to endoscopic colostomy-induced fecal peritonitis [24, 25, 35, 38]. LAPEC showed a technical success rate of 95%, which exceeds the rates reported for endoscopic cecostomy [36]. In critically ill and fragile patients, however, the endoscopic route might be preferred to avoid surgery and extensive sedation [12].

PEC offers several advantages over surgery. Most importantly, general anesthesia can be avoided. Furthermore, the presence of a cecostomy tube prevents stoma stenosis, and the tube is reversible without a need for second operation, although a procedure is still required. Once in place, the PEC tube may be left open for venting. An antegrade enema with polyethylene glycol solution can also be given after initial resolution of ACPO to decrease the risk of recurrence [12, 13, 44]. The tube may be removed once bowel movements are restored and underlying disease leading to ACPO has been treated [12]. Contraindications to the procedure are mechanical obstruction, failure of transillumination, presence of intestinal ischemia, abdominal wall infection, ascites, uncorrected coagulopathy, and sepsis [23, 45].

In our experience limited to three patients, the understanding of the procedure and handling of the PEC system was very intuitive. The learning curve was short, and it rendered the procedures smooth and deliberate, without any adverse events recorded. The placement of PEC was successful and resulted in clinical improvement in all cases (Table 2). One patient (case 2) had to have the colostomy tube placed proximally in the ascending colon because pathologic findings in the cecum made it unfeasible for placement. This was attributed to trophic changes of the cecal mucosa, knowing that some degree of right-sided colon ischemia may be present in approximately 10% of ACPO patients at the time of colonoscopy [13]. Tube removal and fistula closure were uneventful in the single patient in our series (case 3). The tube was removed with colonoscopy assistance. There have been reports of PEC removal by direct traction, although this should be avoided because it could result in cecal tearing or cecostomy tract disruption [15, 16].

The limitations of our study are the small number of patients and its retrospective nature. Yet it reflects the rarity of PEC performance in current practice, and it represents the only series describing the use of PEC in our country to date. Furthermore, our study contributes valuable data to the entire cohort of patients undergoing such a procedure, potentially enhancing the robustness of reporting in the field. Importantly, our manuscript is also accompanied by an illustrative video showcasing the refined PEC technique that had been successfully used in all our patients, providing a recent perspective on PEC placement adhering to the newly published recommendations. For instance, it demonstrates the fixation of the colon to the abdominal wall using 3 T-anchors, a practice that might have been overlooked in earlier studies but is now considered a recommended approach [12]. All preceding series were conducted prior to the availability of dedicated guidelines*.*

## Conclusion

While most patients with ACPO are managed successfully with conventional measures, a subset remains therapeutically challenging. This shows the importance of raising awareness of the entire scope of management options, especially with the increasing numbers of frail octogenarians and nonagenarians. The availability of guideline resources now offers comprehensive guidance for informed decision-making and the procedural aspects.

The results of our experience are in line with previous reports and suggest that PEC may become a very useful tool in the armamentarium of modalities utilized to treat ACPO. Albeit certainly not devoid of adverse events, it offers an alternative for selected patients who would otherwise linger through repeated hospitalizations, multiple decompressive colonoscopies, and for whom surgery represents a disproportionate risk. Nonetheless, large and prospective trials are required in order to truly quantify advantages, if any, over existing treatment strategies, especially in terms of efficacy and safety.

### Supplementary Information

Below is the link to the electronic supplementary material.

Percutaneous endoscopic cecostomy placement – endoscopic and external viewpoints. Supplementary file1 (MP4 241620 kb)

Supplementary file2 (PDF 104 kb)
